# Ulcerated Palatal Mass

**DOI:** 10.1111/odi.70194

**Published:** 2026-07-18

**Authors:** Cintia Mussi Milani, Luiz Augusto Besciak Filho, Marja Cristiane Reksidler, Rafaela Sávio Melzer, Liliane Roskamp, Maria Angela Naval Machado

**Affiliations:** ^1^ Faculty of Dentistry Universidade Tuiuti Do Paraná Paraná Brazil; ^2^ Hospital Erasto Gaertner Curitiba Paraná Brazil; ^3^ Private Practice Paraná Brazil; ^4^ Faculty of Dentistry Universidade Federal Paraná Paraná Brazil

## 1 Case Report

A 39‐year‐old woman presented to an oral medicine clinic complaining of “a swelling in my mouth that appeared 10 years ago and is now interfering with my ability to eat.” She reported that, at that time, her orthodontist had noticed a lesion in the area of the maxillary right molars and recommended a biopsy, but since it was asymptomatic, she did not return for treatment.

Medical history revealed that the patient was a heavy smoker, under continuous medication for anxiety, and extremely fearful of dental procedures. Extraoral examination showed no abnormalities. Intraoral evaluation revealed a large pedunculated nodular mass on the palate with an ulcerated surface, firm on palpation and asymptomatic, extending from the anterior portion of the hard palate to the oropharyngeal region (Figure 1). Oral hygiene was poor, with multiple carious lesions, generalized calculus, and several teeth showing advanced mobility.

**FIGURE 1:**
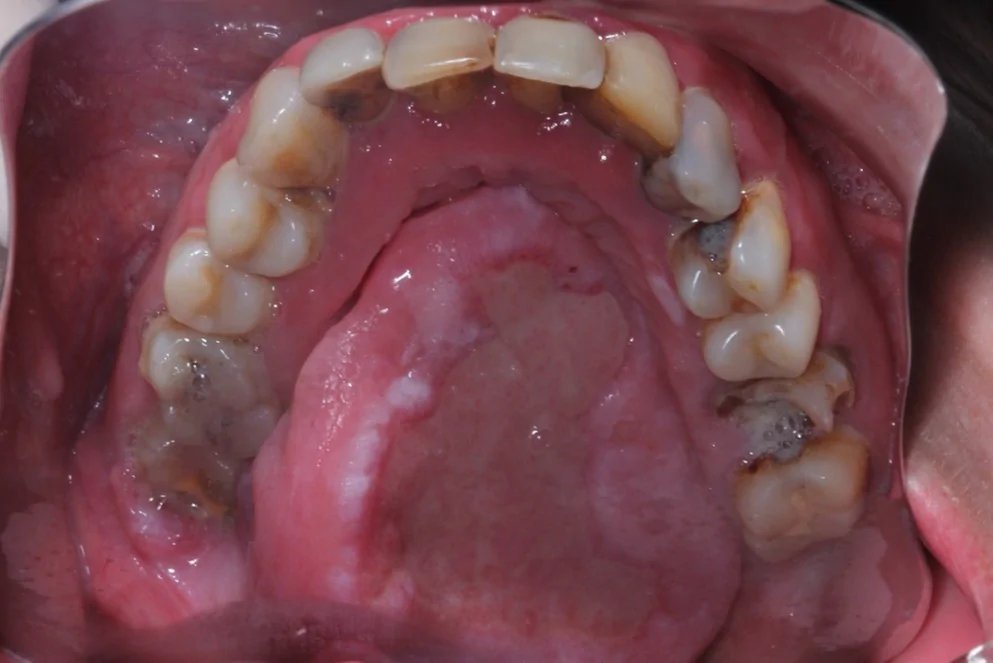
Ulcerated nodular mass extending from the anterior region of the palate to the oropharynx.

## 2 What is Your Diagnosis?

- Pyogenic granuloma
- Peripheral giant cell granuloma
- Peripheral ossifying fibroma
- Mucoepidermoid carcinoma

## 3 Diagnosis

The diagnosis is peripheral ossifying fibroma (POF) is a benign reactive lesion, commonly located on the gingiva and characterized by the proliferation of fibroblasts and the presence of calcifications or bone deposits within the lesion (Baneckova and Agaimy 2020). Its development is generally associated with chronic irritants such as biofilm, dental calculus, irregular restorations, and ill‐fitting prostheses (Lakkam et al. 2020). It is more commonly observed in women between the second and fourth decades of life (Lázare 2019).

Clinically, POF presents as an asymptomatic, slow‐growing nodule that may be pedunculated or sessile, with normal coloration and an intact surface (Baesso et al. 2023); the ulcerated surface exhibited in the present case is less common. The lesion predominantly affects the anterior maxilla (Lázare 2019; Franco‐Barrera 2016; Cavalcante 2022); involvement of the posterior region is infrequent (Mokrysz et al. 2021; Silva 2024). An intraoral photograph from the patient's orthodontic records, taken 10 years earlier, showed the onset of the lesion on the buccal surface of the maxillary right second molar (Figure 2). Cone‐beam computed tomography is recommended to assess the extent of the lesion and identify internal calcifications, a common finding in POF (Figure 3) (Baneckova and Agaimy 2020). Other reactive lesions, such as pyogenic granuloma, fibrous hyperplasia, and peripheral giant cell lesion, should be considered in the differential diagnosis (Lakkam et al. 2020; Baesso et al. 2023).

**FIGURE 2:**
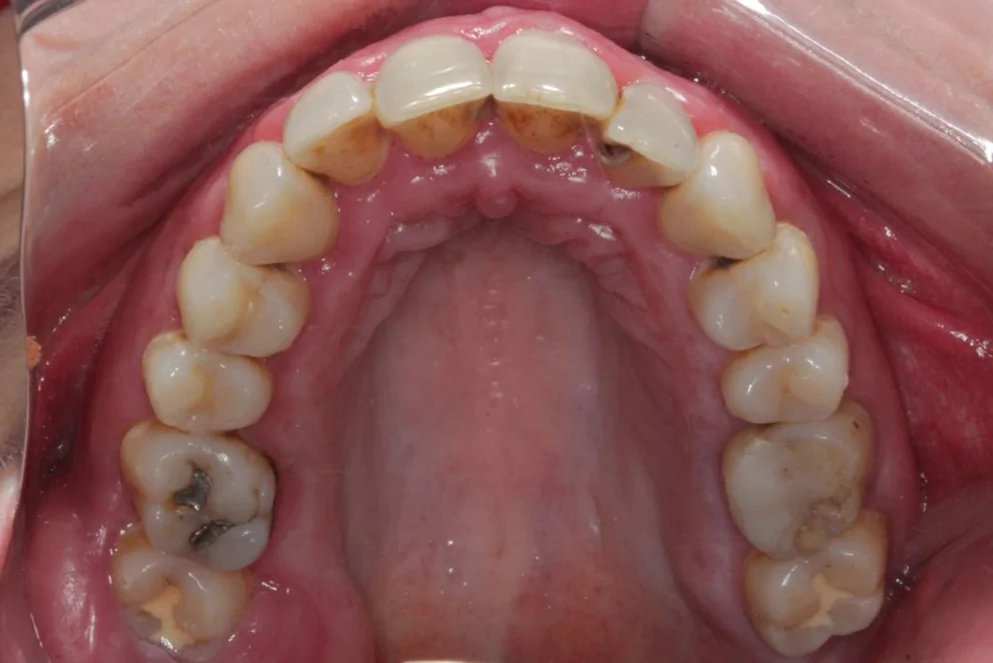
Photograph taken 10 years earlier, showing the onset of the lesion on the buccal surface of the maxillary right second molar.

**FIGURE 3:**
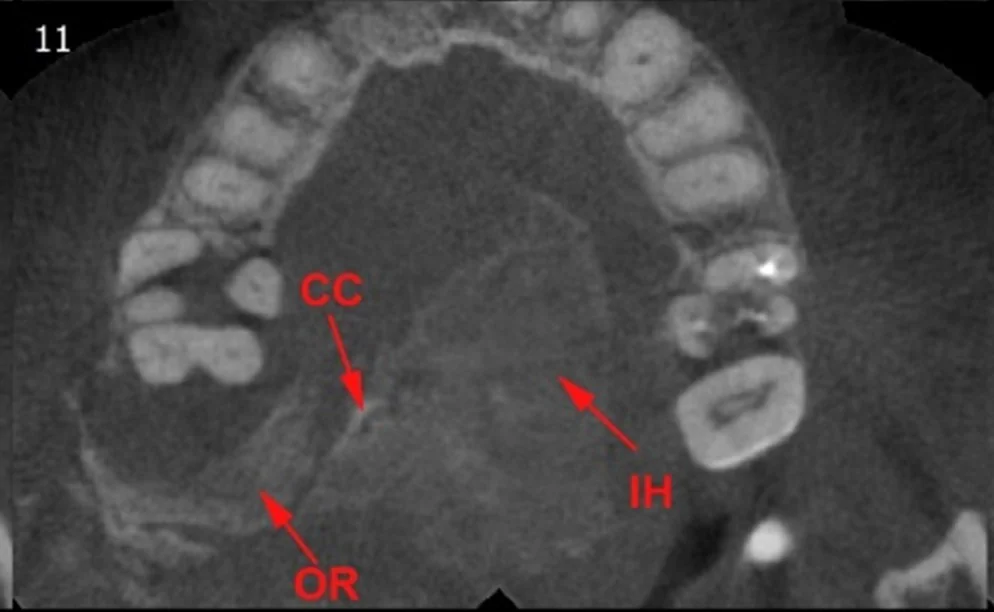
An axial slice of the cone‐beam CT scan reveals a well‐defined, extensive hyperdense image (IH) with the appearance of a pedunculated exophytic lesion, located in the region of the upper right first and second molars. The adjacent bone tissue exhibits reactive characteristics (OR). The lesion extends into the tuberosity and soft palate region and contains hyperdense areas consistent with internal calcifications (CC).

Definitive diagnosis relies on histopathological analysis, which typically reveals bony trabeculae and dystrophic calcifications (Figure 4) (Lázare 2019; Franco‐Barrera 2016; Cavalcante 2022; Shrestha et al. 2021). Treatment of POF consists of local surgical excision associated with the elimination of local irritants to minimize recurrence (Shrestha et al. 2021). In this case, due to the lesion's extensive size, surgery was performed in a hospital setting under general anesthesia after informed consent. Comprehensive dental treatment, including periodontal scaling, restorative procedures, and oral hygiene instruction, was also performed.

**FIGURE 4:**
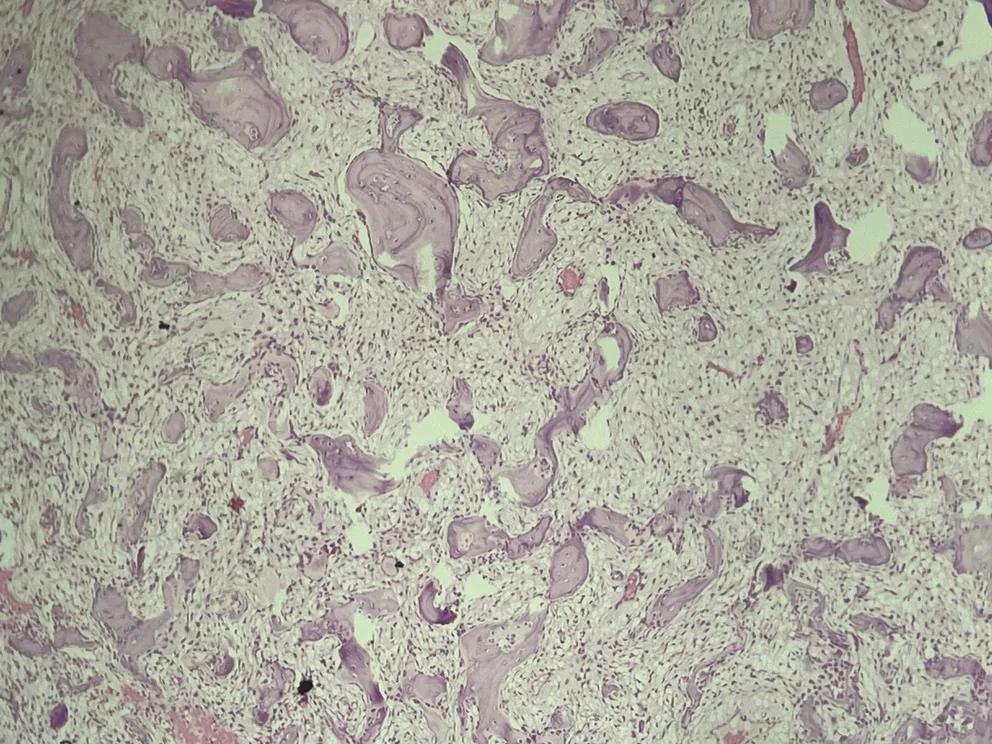
Photomicrograph showing cellularised connective tissue with fibroblast proliferation and immature bone trabeculae (HE, 10×).

Despite being easily visible, the absence of pain and the patient's dental phobia led to delayed care, allowing progression of the lesion and functional impairment. This case highlights the importance of raising public awareness about seeking early dental evaluation at the first sign of any abnormality, whether symptomatic or not. Early intervention can prevent easily manageable lesions from reaching substantial dimensions, thereby reducing morbidity and treatment costs.

## 4 Outcome

The intraoperative and postoperative courses were uneventful, and the 2‐year follow‐up showed no signs of lesion recurrence.

## Author Contributions

**Cintia Mussi Milani:** writing – review and editing, project administration. **Luiz Augusto Besciak Filho:** writing – original draft. **Marja Cristiane Reksidler:** conceptualization, methodology. **Rafaela Sávio Melzer:** investigation, resources. **Liliane Roskamp:** investigation, visualization. **Maria Angela Naval Machado:** supervision.

## Conflicts of Interest

The authors declare no conflicts of interest.

## Data Availability

The data that support the findings of this study are available on request from the corresponding author. The data are not publicly available due to privacy or ethical restrictions.
